# Cardiac output and cardiac index measured with cardiovascular magnetic resonance in healthy subjects, elite athletes and patients with congestive heart failure

**DOI:** 10.1186/1532-429X-14-51

**Published:** 2012-07-28

**Authors:** Marcus Carlsson, Ruslana Andersson, Karin Markenroth Bloch, Katarina Steding-Ehrenborg, Henrik Mosén, Freddy Stahlberg, Bjorn Ekmehag, Hakan Arheden

**Affiliations:** 1 Department of Clinical Physiology and Nuclear Medicine, BFC, Skåne University Hospital, Lund University, Lund, SE-22185, Sweden; 2 Philips Healthcare, Lund, Sweden; 3 Department of Radiation Physics, Lund University, Lund, Sweden; 4 Norrtalje Hospital, TioHundra AB, Norrtalje, Sweden and Lund University, Lund, Sweden

**Keywords:** Cardiac output, Heart failure, Left ventricle, Cardiovascular magnetic resonance

## Abstract

**Background:**

Cardiovascular Magnetic Resonance (CMR) enables non-invasive quantification of cardiac output (CO) and thereby cardiac index (CI, CO indexed to body surface area). The aim of this study was to establish if CI decreases with age and compare the values to CI for athletes and for patients with congestive heart failure (CHF).

**Methods:**

CI was measured in 144 healthy volunteers (39 ± 16 years, range 21–81 years, 68 females), in 60 athletes (29 ± 6 years, 30 females) and in 157 CHF patients with ejection fraction (EF) below 40% (60 ± 13 years, 33 females). CI was calculated using aortic flow by velocity-encoded CMR and is presented as mean ± SD. Flow was validated in vitro using a flow phantom and in 25 subjects with aorta and pulmonary flow measurements.

**Results:**

There was a slight decrease of CI with age in healthy subjects (8 ml/min/m^2^ per year, r^2^ = 0.07, p = 0.001). CI in males (3.2 ± 0.5 l/min/m^2^) and females (3.1 ± 0.4 l/min/m^2^) did not differ (p = 0.64). The mean ± SD of CI in healthy subjects in the age range of 20–29 was 3.3 ± 0.4 l/min/m^2^, in 30–39 years 3.3 ± 0.5 l/min/m^2^, in 40–49 years 3.1 ± 0.5 l/min/m^2^, 50–59 years 3.0 ± 0.4 l/min/m^2^ and >60 years 3.0 ± 0.4 l/min/m^2^. There was no difference in CI between athletes and age-controlled healthy subjects but HR was lower and indexed SV higher in athletes. CI in CHF patients (2.3 ± 0.6 l/min/m^2^) was lower compared to the healthy population (p < 0.001). There was a weak correlation between CI and EF in CHF patients (r^2^ = 0.07, p < 0.001) but CI did not differ between patients with NYHA-classes I-II compared to III-IV (n = 97, p = 0.16) or patients with or without hospitalization in the previous year (n = 100, p = 0.72). In vitro phantom validation showed low bias (−0.8 ± 19.8 ml/s) and in vivo validation in 25 subjects also showed low bias (0.26 ± 0.61 l/min, QP/QS 1.04 ± 0.09) between pulmonary and aortic flow.

**Conclusions:**

CI decreases in healthy subjects with age but does not differ between males and females. We found no difference in CI between athletes and healthy subjects at rest but CI was lower in patients with congestive heart failure. The presented values can be used as reference values for flow velocity mapping CMR.

## Background

Cardiac output (CO) is one of the most important physiological parameters as it directly and proportionally reflects the metabolism of the entire organism. CO is the sum of the systemic flow per minute and calculated by the product of stroke volume and heart rate. The ability of the body to adapt to increased workload and hence metabolism is the result of the ability of the heart to increase heart rate and stroke volume [1]. CO indexed to body surface area (BSA) or cardiac index (CI), is an important clinical parameter used in the assessment of patients with heart disease as well as critically ill patients and patients under anesthesia. CI is also of interest in pharmacological studies [2] and the definition of forward failure in patients with heart failure is a decrease in CI. In clinical cardiology, cardiac output is determined when quantifying intracardiac shunts and during right heart catheterization to determine pulmonary resistance in cases of pulmonary hypertension.

Most available methods for quantifying CI are hampered by the invasive approach and/or the low accuracy and precision [2-6]. Invasive methods include Fick’s principle, thermodilution and dye dilution techniques and non-invasive techniques Doppler measurements and bio-impedance. Cardiovascular magnetic resonance (CMR) offers non-invasive measurements of flow with high accuracy and precision [6,7] and CI can therefore be directly quantified using aortic flow quantification. Previous CI reference values for CMR are based on volumetry measurements but no reference values for CMR using phase contract (PC) flow velocity mapping has been presented.

Flow measurement is recommended in clinical CMR protocols of patients with congenital and/or valvular heart disease [8]. However, it is not known to what degree CI is reduced in patients with heart failure or if athletes show higher CI at rest compared to normal subjects. Furthermore, the relationship between age and CI in normal physiology is not fully established.

Therefore, the main aims of this study were to establish if CI decreases with age and to compare CI in healthy subjects to athletes and patients with heart failure. The presented CI values may also be used as reference values.

## Methods

### Patient population and study design

The study was approved by the local ethics committee. Written informed consent was either obtained or waived by the ethics committee. BSA was quantified according to the Mosteller formula [9].

#### Healthy subjects

144 volunteers aged 21 to 81 years (39 ± 16 years, 68 females) were prospectively included in the study. Age did not differ between females (38 ± 16 years) and males (40 ± 15 years, p = 0.37). All subjects had a normal electrocardiogram (ECG), no history of cardiovascular disease, no cardioactive medication and a blood pressure (BP) of ≤ 140/90 at the time of inclusion or at the time of the magnetic resonance (MR) examination. All healthy subjects had a BMI ≤ 30.

#### Athletes

60 professional athletes with high aerobic capacity (at least 10 hours aerobic training per week) were prospectively included. The athletes were active at a professional level in triathlon, swimming, handball or soccer. Female athletes (n = 30) and male athletes (n = 30) had the same age range and mean ± SD age,19-44 (29 ± 6) years. Age and gender matched controls (n = 60) were selected for comparison of CI between groups. The age and gender distribution of athletes were: up to 29 years 15 males and 25 females; 30–39 years 14 males and 5 females; 40–49 years 1 male.

#### Patients with heart failure (HF)

157 patients from the age of 24 to 86 years, (60 ± 13 years, 33 females) with left ventricular ejection fraction ≤ 40% were recruited retrospectively from patients who had undergone CMR. Patients with more than mild mitral regurgitation (>20% regurgitant fraction), aortic regurgitation or aortic peak velocity >3.0 m/s were not included. Available medical records were reviewed and data was available in 97 patients on New-York Heart Association (NYHA) classification [10] and in 100 patients on the frequency of hospitalization during the last year. The age and gender distribution of patients were: up to 29 years 3 males and 2 females; 30–39 years 4 males and 5 females; 40–49 years 12 male and 0 females, 50–59 years 42 males and 7 females; above 60 years 63 males and 19 females.

### CMR

All subjects were imaged in the supine position with flow quantification of the ascending aorta during free breathing. Two CMR-scanners were used, a) 1.5 T Magnetom Vision (Siemens, Erlangen, Germany) and b) Philips Achieva (Philips, Best, the Netherlands). Blood flow was measured through a transversal plane at the height of the pulmonary bifurcation perpendicular to the ascending aorta with ECG-triggered phase-encoded velocity-mapping sequences. Typical imaging parameters for Siemens Vision were: echo time 5 ms, slice thickness 8 mm, velocity encoding factor 150 cm/s, time resolution typically 40 ms and gradient strength 25mT/m. Velocity information was acquired by prospective ECG-triggering. Typical imaging parameters for Philips Achieva were: echo time 6 ms, slices thickness 6 mm, velocity encoding 200 cm/s, time resolution typically 30 ms, acquired by retrospective ECG-triggering. The gradients for Philips Achieva was set to “regular mode” imposing limits on the maximal gradient strength and slew rate used. A detailed description of the velocity mapping technique has been published previously [11,12]. Cine images of the left ventricle was performed in the short axis view for left ventricular function.

### In vitro and in vivo validation

All in vitro and in vivo validation were performed at the Philips Achieva scanner as we previously have performed and published phantom and in vivo validation for the Siemens scanner [7]. A flow phantom composed of two plastic tubes, connected to give flow in both directions simultaneously, was used. The tubes were surrounded by static material. Water, doped with the clinically used contrast agent, was pumped through the tubes at four different flow rates and was assessed with the same flow quantification sequence as used for the in vivo data. Timer and beaker quantification of flow was used as reference. Aortic and pulmonary flows were measured for in vivo validation in 25 subjects and bias according to Bland-Altman were calculated.

### Image analysis

**Figure 1:**
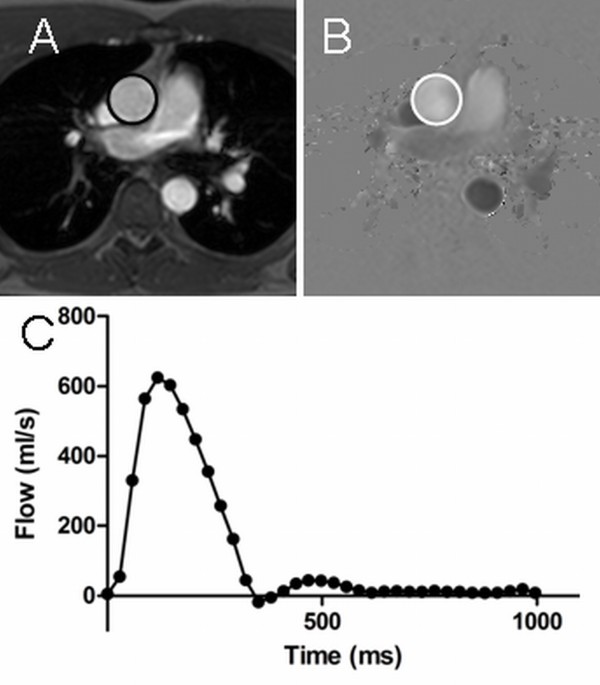
**CMR quantification of cardiac output.** The modulus image (**A**) is used for anatomical delineation of the aorta (black circle) and measurement is performed in the corresponding phase image (**B**). Cardiac output can be calculated by quantifying stroke volume as the integral of the resulting flow curve (**C**) and multiplying with heart rate.

The ascending aorta was outlined in the anatomic images for each time point with flow calculation performed in the corresponding velocity-encoded phase images (Figure 1). The average flow velocity (cm/s) was multiplied by the area of the vessel (cm^2^) to obtain flow (ml/s) at each time point. Flow throughout the cardiac cycle was integrated over one cardiac cycle to obtain the stroke volume (SV). Cardiac output (CO) was determined as SV multiplied by heart rate (SV·HR), and Cardiac index (CI), as CO divided by body surface area (BSA).

Flow was measured using Segment 1.4 (http://segment.heiberg.se) [13]. Two independent observers analyzed the flow data from 20 controls to calculate interobserver variability. Ejection fraction was calculated for all patients using manual outlining of the endocardial contours in the end-diastolic and end-systolic phases.

### Statistical analysis

GraphPad Prism 5.02 (GraphPad Software Inc.) was used for statistical calculations. Continuous variables are presented as mean ± SD. Limits of agreement defined as the mean ± 2SD for CI was calculated within each age group. The Mann–Whitney test was used to differentiate between CI, HR and SV in healthy subjects and patients with HF and athletes as well as between male and females and CI at different age groups. Two-way ANOVA was used to analyze variations in CI due to age and gender. Linear regression analysis was performed for CI, HR and SV vs. age and CI vs. ejection fraction as well as for the in vitro experiments. CI in patients with different NYHA class (I-II vs. III-IV) and the need for hospitalization the previous year was compared using the Mann–Whitney test. Interobserver variability, in vivo and in vitro validation were calculated according to Bland-Altman analysis as the mean difference between the measurements ± SD.

## Results

### Cardiac index in healthy subjects

**Figure 2:**
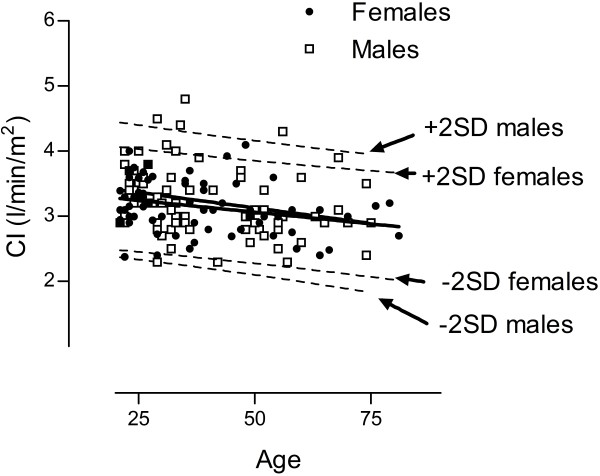
**Cardiac index in healthy subjects declined with age similarily in males (open squares) and females (filled circles).** The linear regression lines are shown in solid lines and the 95% confidence interval with broken lines. The relationship between CI and age was similar in males (y = −0.0095age + 3.275, r^2^ = 0.08, p = 0.02) and females (y = −0.0072age + 3.423, r^2^ = 0.08, p = 0.02).

Cardiac index in healthy subjects did not differ between males (3.2 ± 0.5 l/min/m^2^) and females (3.1 ± 0.4 l/min/m^2^, p = 0.64) but there was a slight decrease of CI with age (8 ml/min/m^2^ per year, r^2^ = 0.07, CI = −0.008xage + 3.511, p = 0.001), Figure 2. Similarly, age (p = 0.01) but not gender (p = 0.57) was a significant variable for CI in the two-way ANOVA analysis. The slight decrease of CI with age was explained by a decrease in SV with age (r^2^ = 0.06, SV = −0.27xage + 106.4, p = 0.004). Heart rate did not change with age (p = 0.87). The mean ± SD of CI and limits of agreement for healthy subjects according to age and gender is listed in Table 1. The minimum CI found in an individual was 2.3 l/min/m^2^. The interobserver variability of cardiac output measurements was 0.2 ± 0.2 l/min or 3 ± 4%.

**Table 1:** Cardiac index and age adjusted reference values

**Age (years)**	**Males (n)**	**Females (n)**	**CI (l/min/m**^**2**^**)**	**Reference values**	**p vs. CI of −29 years**
−29	25	30	3.3 ± 0.4	2.5-4.1	NA
30-39	20	12	3.3 ± 0.5	2.3-4.3	ns.
40-49	8	10	3.1 ± 0.5	2.1-4.1	ns.
50-59	13	9	3.0 ± 0.4	2.2-3.8	<0.001
60-	10	7	3.0 ± 0.4	2.2-3.8	0.007

### Cardiac index in athletes vs. healthy subjects

**Figure 3:**
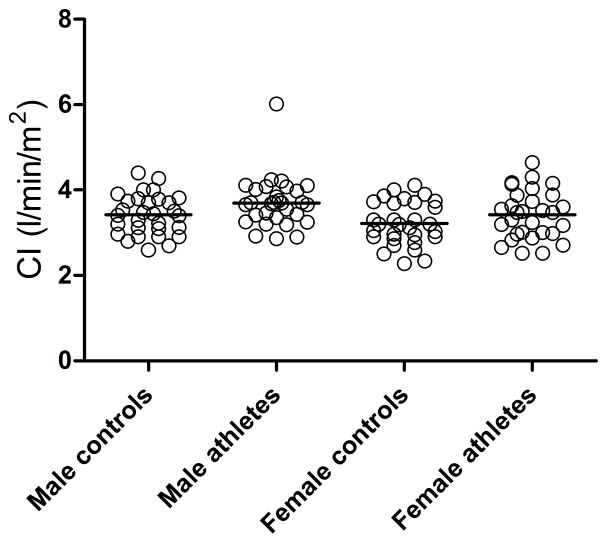
**There were no significant differences in cardiac index of male and female athletes compared to age and gender matched controls.** One outlier in the male athlete group was seen and the stroke volume (174 ml) and heart rate (68/min) were confirmed on cine-CMR.

Cardiac index in female (3.4 ± 0.6 l/min/m^2^) and male athletes (3.7 ± 0.6 l/min/m^2^) did not differ significantly (p = 0.09, Figure 3). There was no difference in CI between athletes and age-matched healthy subjects in either females (p = 0.20) or males (p = 0.07). Athletes had higher stroke volume index quantified on PC-CMR (66 ± 7 ml/m^2^ for males and 60 ± 9 ml/m^2^ for females) compared to age and gender matched healthy subjects (54 ± 6 ml/m^2^ for males and 50 ± 7 ml/m^2^ for females, p < 0.001 for both). On the other hand, heart rates were lower in athletes (males 56 ± 7/min and females 58 ± 9/min) compared to controls (males 64 ± 10/min and females 64 ± 12/min, p = 0.003 and p = 0.01, respectively).

### Cardiac index in patients vs. healthy subjects

**Figure 4:**
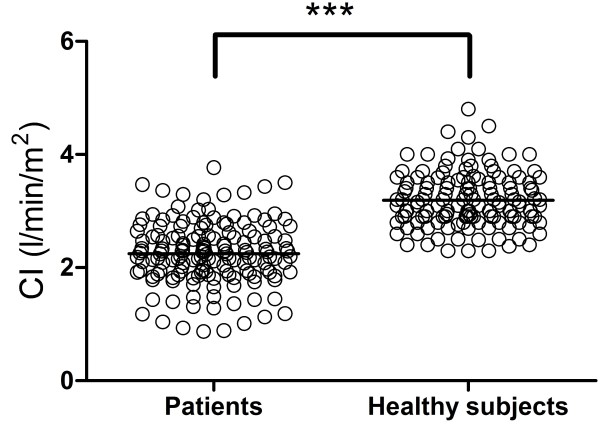
Cardiac index in patients were lower compared to healthy subjects (*** p < 0.001) although there was a large overlap.

**Figure 5:**
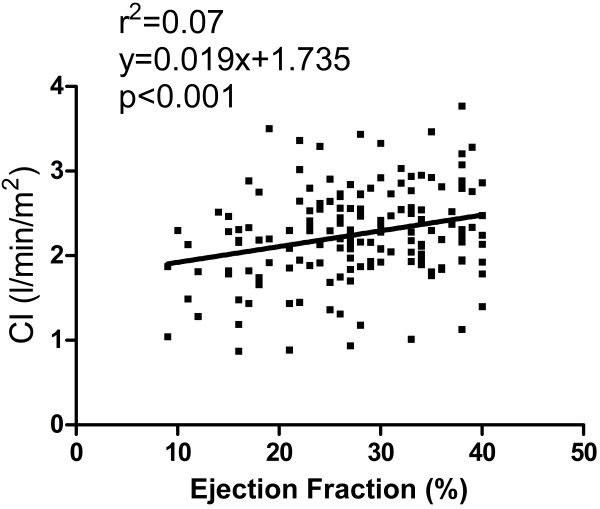
There was a weak correlation of cardiac index with ejection fraction.

**Figure 6:**
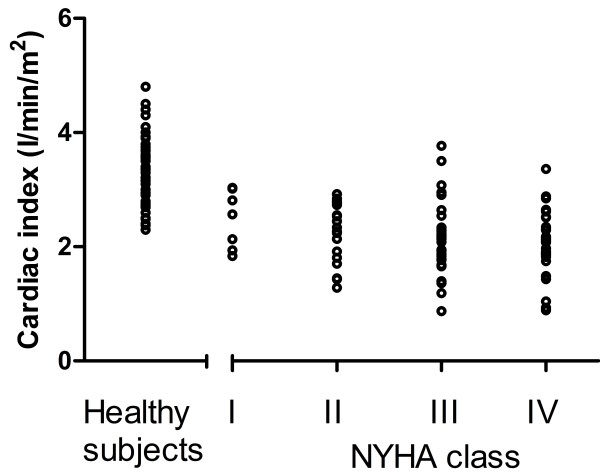
**Patients with NYHA class III and IV did not have lower CI compared to patients with NYHA class I and II (p = 0.16).** CI of healthy subjects are showed for comparison.

CI in patients with congestive heart failure (2.3 ± 0.6 l/min/m^2^) was lower compared to the healthy population (3.2 ± 0.5 l/min/m^2^, p < 0.001), although there was a large overlap (Figure 4) with 49% of patients´ CI within mean ± 2SD of healthy subjects´ CI. Patients had a lower effective SV index (32 ± 9 ml/m^2^) compared to healthy subjects (51 ± 7 ml/m^2^, p < 0.001) and higher heart rate (73 ± 15/min and 64 ± 9/min respectively, p < 0.001). The subset of patients with normal CI had lower SV index (36 ± 7 ml/m^2^, p < 0.001) and higher heart rate (76 ± 15, p < 0.001) compared to healthy subjects but higher SV index and heart rate compared to patients with decreased CI (28 ± 8 ml/m^2^, p < 0.001 and 70 ± 15/min, p = 0.006, respectively). In patients, CI had a weak relationship with decreasing EF (r^2^ = 0.07, p < 0.001, equation CI = 0.02xEF + 1.7), Figure 5. There was no significant correlation with age in the patient population (r^2^ = 0.004, p = 0.56). Cardiac index did not differ between NYHA-classes I-II (2.3 ± 0.5 l/min/m^2^) and NYHA classes III-IV (2.2 ± 0.6 l/min/m^2^, p = 0.16), Figure 6. Neither did CI differ in patients with hospitalizations the previous year (2.2 ± 0.5 l/min/m^2^) vs. patients without hospitalizations (2.3 ± 0.6 l/min/m^2^, p = 0.72).

### In vitro and in vivo validation

**Figure 7:**
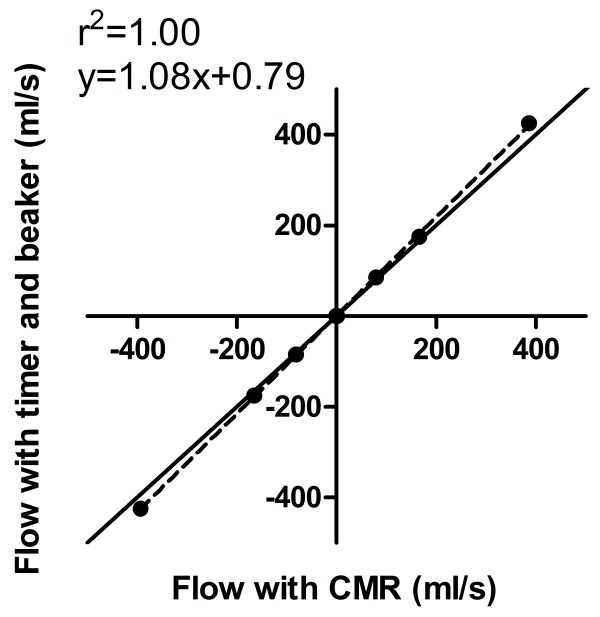
Flow data from in vitro phantom experiments showed an excellent correlation between flow measured with timer and beaker and CMR.

Phantom measurements showed an excellent correlation (r^2^ = 1.00, y = 1.08x + 0.79, p < 0.001) and Bland-Altman analysis showed a low bias (−0.8 ± 19.8 ml/s) between flow as measured by timer and beaker and by MR, Figure 7. The bias according to Bland-Altman analysis between pulmonary and aortic flow was 0.26 ± 0.61 l/min or 4 ± 8%. This results in a QP/QS of 1.04 ± 0.09.

## Discussion

This study has confirmed that CI decreases with age, probably caused by a decreased level of metabolism. There were no differences between CI in males and females in healthy subjects and athletes had similar CI values to healthy subjects. CI was found to be lower in patients with heart failure compared to healthy subjects, although 49% of the heart failure patients fall within the normal ranges of CI. Finally, there was a weak correlation of CI and EF but no differences were found in CI between patients with NYHA-class I-II and III-IV or the absence/presence of hospitalization during the last year.

### Normal CI values and variation with age

This study showed a decrease of CI with age (8 ml/min/m^2^) which is in line with lower stroke volumes in older subjects found in reference values of cine CMR [14,15]. Metabolism decreases with age, especially in sedentary individuals [16,17] and this is the most likely explanation to the decrease for CI with age. We did not find any gender difference in CI which is in line with our previously published normal values for gradient-echo cine CMR images [15] where no gender differences were seen in SV adjusted for BSA. Maceira et al., however, did find higher SV corrected for BSA in males compared to females [14]. The difference between our studies are not clear and further studies in other cohorts comparing SV and CI between gender would therefore be of interest. The mean CI values in this study, 3.1 l/min/m^2^ for women and 3.2 l/min/m^2^ for men, are the same mean values previously described by our group [7].

### Cardiac index in athletes

CI at rest was found to be similar in athletes and subjects with regular exercise levels already in the 1960´s using invasive techniques [1] but this is the first study to show that the same reference values can be used for CI in athletes as for other healthy subjects. The lack of a difference in CI between normals and elite athletes underscores that cardiac CI is primarily dependent on the basal metabolism. The higher SV and lower HR in athletes compared to controls reiterates the higher reserve capacity for increasing CI through HR increase during stress in athletes. Even if there are no differences in CI between healthy volunteers and elite athletes at rest, the differences become clear during exercise [1]. The main difference induced by training is that stroke volume at peak exertion increases [18]. This is in line with the findings from our previous CMR study of 60 healthy volunteers and 71 elite athletes demonstrating that VO_2max_ is proportional to total heart volume [19]. The larger total heart volumes in athletes results in a possibility of generating larger cardiac output due to higher stroke volume at similar heart rate.

### Cardiac index in heart failure

Half of the patients had CI within normal limits and this was obtained through a higher heart rate although stroke volume was decreased compared to healthy subjects. The finding that 49% of the patients with heart failure were within normal limits of CI at rest shows that CI on its own is not useful as an indicator of heart failure in patients that fall within normal limits. An earlier study has suggested a method to differentiate these patients from healthy subjects by relating VO_2max_ to total heart volume (THV) [20]. In that study, patients with increased THV fell below normal subjects in VO_2max_ capacity.

Quantification of CI is primarily performed in the clinical management of heart failure when assessing pulmonary vascular resistance and shunt lesions. Medication of heart failure often aims to increase the CI and CMR could play an important role when assessing new therapies for heart failure. The high accuracy with CMR would lower the number needed for detection of therapeutic effect.

### Measurements of CI with CMR

Flow quantification with PC-CMR has been extensively validated and found to have high accuracy in large [7,21-23] and small [24,25] vessels. Studies with segmented sequences in modern scanners with shorter bore have shown problems with phase offsets thay may cause errors in CI measurements PC-CMR [26,27]. The baseline shifts caused by phase offsets can be corrected for by dedicated post-processing or by using phantoms [28]. The transversal image plane for aortic flow used in our study have lower phase offset compared to oblique image planes more proximal to the aortic valve [27]. The CI quantification in this study was done using a non-segmented sequence during free breathing and with the use of a lower gradient mode. Restricting the gradient strength can help to decrease the phase-shift errors (personal communication Dr. M Kouwenhoven, Philips Healthcare, Best, the Netherlands) and the flow results from the validations in this study in phantoms and in vivo showed low bias within the physiological range. Of note, the in vivo validation found a mean QP/QS of 1.04 which is the expected value taking into account a coronary artery flow of 3-5% of cardiac output.

### Limitations

This study was performed in a single center using two different scanners from different vendors. Full medical records could not be obtained in all patients and therefore not all patients were included in the analysis for a possible relationship between CI and NYHA class and presence of hospitalization. Examinations in a CMR scanner may cause psychological stress due to the confined space and examination in a hospital setting. This may have resulted in a higher CI values compared to the CI at complete rest at home.

## Conclusions

CI over a wide age range in healthy subjects are presented and can be used as reference values for CMR. CI decreases in healthy subjects with age but does not differ between males and females. We found no difference in CI between athletes and healthy subjects but CI was lower in patients with congestive heart failure.

## Competing interests

KMB is an employee of Philips Healthcare.

## Authors’ contributions

MC and HA conceived and designed the study and drafted the manuscript. KMB and FS carried out the phantom data collection and analysis. KSE and HM participated in subject enrollment and data collection. RA and BE reviewed the patients charts for clinical data. MC performed the statistical analysis. All authors revised the manuscript during its preparation and have read and approved the final manuscript.
